# Anal phyllodes tumor in a *male* patient: a unique case presentation and literature review

**DOI:** 10.1186/1746-1596-8-49

**Published:** 2013-03-26

**Authors:** Szu-pei Ho, Hui-hwa Tseng, TM King, Philip-C Chow

**Affiliations:** 1 Department of Pathology and Laboratory Medicine, Kaohsiung Veterans General Hospital, Kaohsiung, Taiwan; 2 Department of Colorectal Surgery, Kaohsiung Veterans General Hospital, Kaohsiung, Taiwan; 3 Department of Psychiatry, Kaohsiung Veterans General Hospital, Kaohsiung, Taiwan

**Keywords:** Anogenital mammary-like glands, Ectopic breast tissue, Fibroepithelial neoplasm, Phyllodes tumor, Gynecomastia

## Abstract

**Abstract:**

Lesions of anogenital mammary-like glands are rare, and only 44 female cases have been reported. Herein, we describe a particularly rare case of phyllodes tumor of anogenital mammary-like glands in a 41-year-old male presenting anal bleeding. Papillectomy was performed. The excised tumor was circumscribed in shape, and after it was sliced into sections, it was noted that there were leaf-like slits on the surface of cut side. Under the microscope, the tumor was found to be biphasic, with a bland glandular epithelium and low-to-intermediate cellular stroma, which together created the leaf-like slits. Gynecomastoid hyperplasia was evident at the periphery. The epithelium showed immuno-activity for ER, PR(focal), AR, and GCDFP-15. The stromal cells showed positive staining for CD34 and vimentin. The morphology and immunophenotype were similar to benign phyllodes tumors of breast. To the best of our knowledge, this case report represents the first case of phyllodes tumor of anogenital mammary-like glands with gynecomastoid hyperplasia at the periphery in a male patient. To make a diagnosis, we had to differentiate this lesion from hidradenoma papilliferum of skin appendage, phyllodes tumor of ectopic prostatic tissue, and other tumors of anogenital mammary-like glands analogous to the breast tumor (e.g., fibroadenoma phyllodes, periductal stromal sarcoma, and spindle cell carcinoma). While gynecomastia of male breast is usually a result of hormone imbalance, our patient’s tumor did not seem to be related to peripheral hormone status in the anogenital mammary-like glands. Nevertheless, because hormone imbalance has been strongly related to male breast cancer, hormone levels may need to be followed in male patients who have this rare malady.

**Virtual slides:**

The virtual slide(s) for this article can be found here: http://www.diagnosticpathology.diagnomx.eu/vs/1509145815899177

## Background

Ectopic breast tissue in the vulva was first identified by Hartung in 1872, and it has long been considered caudal remnants of the milk ridges, though mammary type tissue has been reported to be a normal constituent of the anogenital area (1991) [1]. Today such tissues in the anal area are called anogenital mammary-like glands (AGMLG). There have been reports of lesions arising in AGMLG that resemble breast neoplasms, including benign or malignant, epithelial and/or stromal neoplasms. Phyllodes tumor or other fibroepithelial tumors of AGMLG is extremely rare and has previously been found in females exclusively [2-4]. Herein, we present the first case of a male patient found to have low-grade phyllodes tumor of AGMLG. Gynecomastoid hyperplasia of AGMLG was also identified at periphery of tumor. Because all fibroadenomas of the male breast have been found with concurrent gynecomastia and patients with these lesions have clear hormone imbalances [5], we assumed that the phyllodes tumor or other fibroepithelial neoplasms of AGMLG with gynecomastoid hyperplasia at the periphery might also suggest possible hormone imbalance. However, peripheral estrogen/androgen ratio was found to be unchanged.

## Case presentation

### Case report

**Figure 1:**
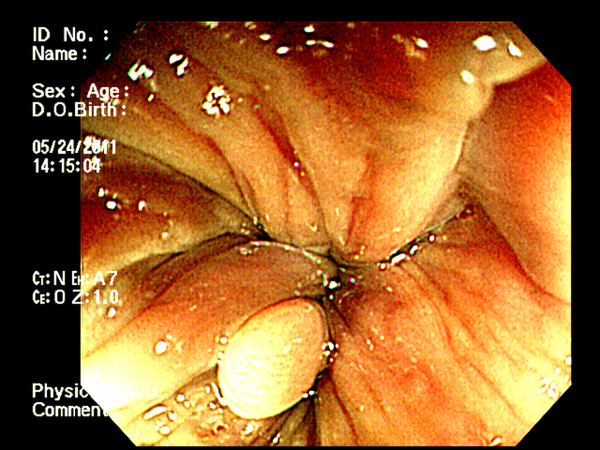
**Colofibroscopic finding. **The endoscopic findings revealed a subepithelial tumor at anal verge.

A 41-year-old Taiwanese male with a previous history of hypertension and major depressive disorder under control with medication came to the outpatient clinic of the Division of Gastroenterology complaining of recent anal bleeding. Physical examination and colon fiberoscopy revealed a subepithelial tumor at anal verge (Figure 1), for which he received a papillectomy.

## Materials and methods

For pathology analysis, representative specimens were fixed in 4% buffered formalin and embedded in paraffin. They were sliced into serial sections (4um) and stained with hematoxylin-eosin. Immunohistochemistry stains were performed using Leica BOND-MAX. We performed immunostaining for: estrogen receptor (Clone 6F11); progesterone (Clone 16); androgen receptor (Clone AR27); gross cystic disease fluid protein 15 (Clone 23A3); vimentin (Clone SRL33); CD34 (Clone QBEnd/10); actin (Clone HHF35); smooth muscle actin(SMA) (Clone alpha sm-1); Ki-67(MIB-1) (Clone GM010); Cytokeratin 7(CK7) (CloneOV-TL 12/30); Prostatic Acid Phosphatase (PSAP) (Clone PASE/4LJ); prostate-specific antigen (PSA) (Clone 35H9); Pan-cytokeratin (Clone AE1/AE3).

## Results

**Figure 2:**
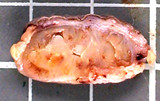
**Gross features. **The tumor appeared circumscribed. The cut sections showed leaf-like slits.

Grossly, the subepithelial tumor measured 2.4 cm at the greatest circumference. It was grey-white, mildly firm, well-circumscribed with a cleft-like appearance (Figure 2). The skin overlying the anal tumor had no remarkable findings.

**Figure 3:**
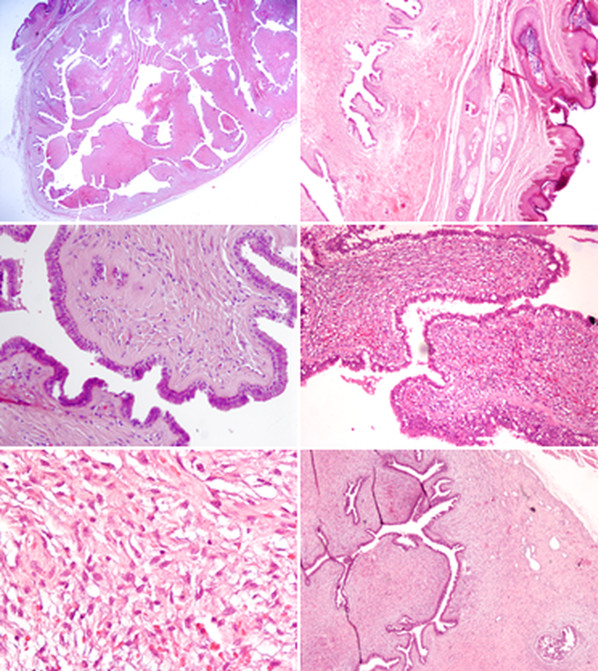
**Microscopic findings. a) **At the low power field, the tumor was found to be well-circumscribed. Leaf-like growth pattern was clearly depicted. **b) **The overlying skin and skin appendage of this tumor showed no remarkable change. **c) **The epithelium was composed of double layers. The stroma showed low cellularity. **d) **However, there is other area of tumor which shows increased stromal cellularity in low-to-intermediate degree. **e) **At high power field, stormal hypercellularity and cell atypism were identified. **f) **At peripheral, gynecomastoid hyperplasia featuring proliferation of both ductal and stromal cells was found.

**Figure 4:**
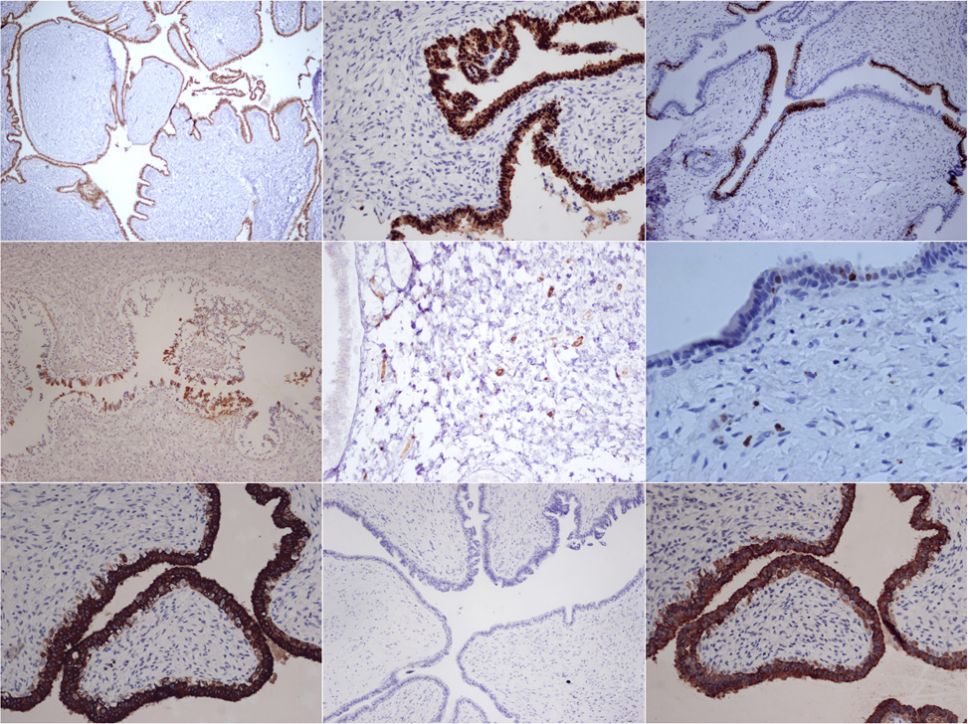
**Immunohistochemistry. a) **ER. **b) **PR.**c) **AR. **d) **GCDFP-15. **e)** CD34. **f) **Ki-67. **g) **CK7. **h) **PSAP. **i) **AE1/AE3.

Microscopically, the circumscribed tumor was composed of the biphasic components of glandular epithelium and stromal component, together forming leaf-like slits (Figure 3a). The glandular epithelium consisted of a luminal ductal epithelium layer and a basal myoepithelial layer. Stroma showed low-to-intermediate cellularity (Figure 3d-e). An increase in Ki-67 proliferative index was found in stromal cells. (Figure 4f). The morphology of the tumor was similar to that of the mammary glands under phyllodes change. At the peripheral of tumor, both ductal epithelium and periductal stroma proliferated without lobular pattern (Figure 3f). The stroma were myxoid or edematous in appearance. These characteristics were similar to those of gynecomastia on the male breast [5].

In the immunohistochemical study, the luminal ductal epithelium showed positive for ER and PR and focal positive for AR and GCDFP-15 (Figure 4a-d). The basal myoepithelial cells showed positive for CK5/6, actin, and SMA. The stromal cells showed positive for CD34 (Figure 4e) and vimentin and negative for actin and SMA.

The patient remained in the hospital for three days and recovery was unremarkable. He was discharged on day 4. As of 20 months post-surgery, there was no local recurrence.

## Discussion

Lesions of AGMLG present as a spectra of morphologic changes synonymous with those in the breast, including lactating adenoma, hidradenoma papilliferum, syringoadenoma papilliferum, fibroadenoma, phyllodes tumor, pseudoangiomatous stromal hyperplasia, extramammary Paget’s disease and other malignancies arising from AGMLG [6]. Benign changes, such as apocrine, oxyphilic or squamous metaplasia, myoepithelial hyperplasia or clear cell metaplasia, lactation-like change and florid epithelial hyperplasia, have also occasionally been identified [6]. To date, fibroepithelial lesions of AGMLG have only been reported in 44 female cases. As far as we know, there has been no report of a case of this tumor of AGMLG in a male patient.

We performed differential diagnoses to exclude possible hidradenoma papilliferum of skin appendage, phyllodes tumor of ectopic prostatic tissue, and tumors of AGMLG which are analogous with the breast neoplasm. Hidradenoma papilliferum can also be found in the anal region [7-9]. However, hidradenoma papilliferum has a more complex papillary structure than the leaf-like pattern of benign phyllodes tumors [10]. In hidradenoma papilliferum, glandular secretion is incapacitated. Because lesions of mammary glands and sweat glands stain for ER and GCDFP-15, immunohistochemistry may not be used to distinguish hidradenoma papilliferum from mammary lesions [8]. Phyllodes tumor has been found in ectopic prostate tissue, mostly in female patients, the most common locations being the vulva, vagina, cervix, urinary bladder and anal canal [11-13]. Gynecomastoid hyperplasia around the main lesion may cause a pathologist to suspect residual normal prostate glands. Mammary glands may show weakly positive for PSA. However, a positive finding of CK7 and negative finding for PSAP may help exclude the possibility of a prostatic origin (Figure 4g-h) [11]. There are other tumors homologous to the breast tumor. One, periductal stromal sarcoma, also has a biphasic growth pattern. However, it does not have the leaf-like appearance of intracanicular growth pattern but rather a solid growth appearance of a pericanalicular pattern [14]. Another such tumor is spindle cell carcinoma. The epithelioid nests of this tumor may merge with spindle stromal elements. Cytokeratin stain can help visualize these tumor cells clearly (Figure 4i) [14]. Still another such tumor is fibroadenoma phyllodes, which is a histological feature similar to that of low-grade phyllodes tumor [15]. It can be difficult to distinguish the two because there is a continuum of morphologic findings. However, if the lesion has well-formed leaf-like slits and the hypocellular stroma appears to the degree that is found in fibroadenoma, it is called fibroadenoma phyllodes [15]. In our case, there was increased stromal cellularity and cell atypia. The proliferative index of stromal cells was found to be increased when viewing the Ki-67 Immunostain (Figure 4f). Together, these characteristics help distinguish phyllodes tumor from fibroadenoma phyllodes. Regardless, in the mammary gland, both fibroadenoma and phyllodes tumor carry risk of local recurrence.

Benign lesions in AGMLG outnumber malignant ones more than they do in the breast, possibly because the anus has more superficial locations [6]. In all patients reported previously, there was no recurrence of the tumor after excision [6]. As of twenty months follow-up, no local recurrence was found in our case. It is unclear whether phyllodes tumor of AGMLG has a better prognosis than phyllodes tumor of the mammary gland. Because it is difficult to predict behavior of this tumor in mammary glands, the possibility of recurrence locally cannot be totally excluded. Likewise, it would be prudent to closely follow-up phyllodes tumor of AGMLG post-surgery.

According to Sandra J. , there is always concurrent gynecomastia in fibroadenoma of breast in men [5]. Both gynecomastia and male breast cancer share hormonal imbalance as a same factor. Exogenous estrogen use in the male patient is evident [5]. Because our case had gynecomastoid hyperplasia and tumor on his AGMLG, we were concerned that he may also have a hormone imbalance as well as gynecomastia or tumor on the breast. However, his hormone levels (prolactin, beta-hCG, testosterone, E2, LH, FSH, T3, T4) were within normal range, and no gynecomastia or tumor was found in breast. These negative findings could be interpreted to suggest that our patient’s phyllodes tumor and gynecomastoid hyperplasia of AGMLG resulted from localized hormone change. Although we found no peripheral hormone change, it might be still wise to monitor hormone levels in other such patients because of the known strong association between hormone level and gynecomastia as well as male breast cancer.

The alterations in ratio of androgen to estrogen may be influenced by many factors [16]. In middle-aged men, changes in this ratio can be caused by testicular or adrenal tumors, hormone-secreting tumors, hormone deficiency, prostate cancer, obesity, liver or renal disease, medical history of disease or drug abuse. Our male patient had normal hormone status (prolactin, beta-hCG, testosterone, E2, LH, FSH, T3, T4), and denied taking any drugs except for those he was taking for hypertension and depression. However, in such patients, it is necessary to take a careful medical and drug-use history as well as perform comprehensive evaluations, including physical examination, imaging, and laboratory evaluations.

## Conclusions

In summary, herein we have introduced the first case of a male patient with a phyllodes tumor in anogenital mammary-like glands. Gynecomastoid hyperplasia of AGMLG was also identified, though no hormone imbalance or breast manifestations were found. We are unsure whether the phyllodes tumor of AGMLG lesion in our male patient was indeed a localized lesion or whether he would develop breast cancer in the future. Further study is needed to establish the clinical significance of this manifestation.

### Consent

Written informed consent was obtained from the patient for publication of this case report and accompanying images. A copy of the written consent is available for review by the Editor-in-Chief of this journal.

## Abbreviations

AGMLG: Anogenital mammary-like glands.

## Competing interests

The authors declare that they have no competing interests.

## Authors’ contributions

HHT conceived the study, participated in its design, coordinated research efforts, and helped draft and edit the manuscript. SPH participated in the pathological analysis and drafted the manuscript. TMK and P-CC contributed to the collection of clinical data. All authors read and approved the final version of the manuscript.
